# Simultaneous Necking and Barreling Deformation Behaviors in Bending of Single-Crystal Gold Micro-Cantilever

**DOI:** 10.3390/ma17164054

**Published:** 2024-08-15

**Authors:** Kazuya Fujita, Kosuke Suzuki, Keisuke Asano, Chun-Yi Chen, Tomoyuki Kurioka, Katsuyuki Machida, Hiroyuki Ito, Yoshihiro Miyake, Masato Sone, Tso-Fu Mark Chang

**Affiliations:** 1 Institute of Innovative Research, Tokyo Institute of Technology, Yokohama 226-8501, Japanchen.c.ac@m.titech.ac.jp (C.-Y.C.); kurioka.t.aa@m.titech.ac.jp (T.K.);; 2 Department of Computer Science, Tokyo Institute of Technology, Yokohama 226-8501, Japan

**Keywords:** single-crystalline gold, bending test, micro-cantilever, deformation behavior, slip system, yield stress, sample geometry effect, MEMS

## Abstract

Necking and barreling deformation behaviors occurred simultaneously during the bending test of a single-crystal gold micro-cantilever (sample A) with the loading direction parallel to the [1-10] orientation and the neutral plane parallel to the [110] orientation. In contrast, for another single-crystal gold micro-cantilever, sample B, with the loading direction aligned parallel to the [0.37 −0.92 0.05] orientation and the neutral plane parallel to the [0.54 0.28 0.78] orientation, predominant slip band deformation was noted. Sample A exhibited activation of four slip systems, whereas sample B demonstrated activity in only a single-slip system. This difference suggests that the presence of multiple slip systems contributes to the concurrent occurrence of necking and barreling deformations. Furthermore, variations in the thickness of the micro-cantilevers resulted in observable strengthening, indicating that the effect of sample size is intricately linked to the geometry of the cross-section, which we have termed the “sample geometry effect”.

## 1. Introduction

Gold materials are increasingly incorporated into electronic devices due to their exceptional corrosion resistance, chemical stability, and conductivity [1,2,3]. Recent advancements have extended the use of gold materials to the movable components of micro-electro-mechanical system (MEMS) devices, facilitating further miniaturization while maintaining or enhancing device performance [4,5,6,7,8]. A critical factor in the functionality of MEMS devices is their sensitivity, which is significantly influenced by Brownian noise [5]. Notably, Brownian noise is inversely proportional to the mass of the movable component within the device. Therefore, the high mass density of gold not only contributes to a reduction in the Brownian noise but also supports the ongoing miniaturization of MEMS devices. This dual benefit is evidenced in MEMS accelerometers, where the utilization of gold materials has been demonstrated to achieve low Brownian noise levels and high sensitivity without compromising the compactness of the device, leveraging gold’s high mass density to optimal effect [4,5].

The mechanical properties of metallic materials are intricately linked to the behavior of dislocations, which are themselves influenced by factors such as crystal orientation [9], grain boundaries [10,11], twins [12], textures [13,14], and more. Notably, barreling deformation is a common observation in compression tests [15], while necking deformation tends to occur in tension tests [16]. Furthermore, when the size of metallic samples is reduced to the micro or sub-micro scale, their mechanical strength is impacted by what is known as the sample size effect [17,18,19,20,21,22,23,24,25]. Various theories have been proposed to elucidate this phenomenon, including the dislocation starvation model [17]. This model suggests that mobile dislocations are more likely to escape to free surfaces than to multiply and interact with other dislocations. Additionally, in scenarios where strain gradients are present within the sample, such as during bending tests, a reduction in sample dimensions can increase the number of geometrically necessary dislocations, thereby contributing to the sample size effect [21]. This effect implies that the mechanical properties of materials at the micro scale can significantly differ from those observed in bulk-sized materials.

Given that components within miniaturized electronic devices often fall into the micro scale or smaller, understanding the mechanical properties of samples at comparable sizes is crucial. To address this need, micro-mechanical testing techniques, such as compression [13,26], tension [27,28], and bending tests [29,30], have been developed for micro-sized samples. These methods are essential for accurately characterizing the mechanical behavior of materials used in small-sized electronic components.

During operation, movable components within a MEMS device are subjected to both compressive and tensile stresses. Therefore, the bending test is recommended as the optimal method for characterizing mechanical properties in the context of MEMS applications. Concerning the sample size effect, it has been observed that the strength of a material exhibits a power-law relationship with its sample’s cross-sectional area [17]. During the bending test, the cross-sectional area exhibits geometric anisotropy. In the case of a cantilever-type sample possessing a rectangular cross-section, the force loading direction is perpendicular to the width of the cantilever and parallel to its thickness. Consequently, alterations in the cross-sectional area, achieved by varying either the width or thickness, result in distinct impacts on the sample size effect. This phenomenon is identified as the sample geometry effect [29].

In this research, we further investigate the impact of sample geometry by utilizing micro-cantilevers made from single-crystal gold to eliminate the influence of grain boundaries [18] on the sample size effect. Furthermore, we confirm the occurrence of both necking and barreling deformations in the single-crystal gold micro-cantilever, and explore the relationship between the activated slip system and this distinctive deformation behavior.

## 2. Materials and Methods

### 2.1. Materials

A piece of commercially available single-crystal gold supplied by Sanwa Kinzoku Co., Ltd., Kyoto, Japan, was used to prepare the sample A micro-cantilever. The Sanwa Kinzoku gold’s top and bottom surfaces were in the {001} plane and the side surfaces were in the {110} plane. The purity of the Sanwa Kinzoku gold was 5N (99.999%). For the sample B micro-cantilever, a piece of commercially available polycrystal gold provided by the Nilaco Corporation (Tokyo, Japan) was used, and the purity was 99.95%. The Nilaco gold underwent heat treatment at 700 °C for 5 h. Following the heat treatment, the Nilaco gold was examined using an electron backscatter diffraction (EBSD) system (BRUKER QUANTAX CrystAlign EBSD system, Tokyo, Japan) equipped in a scanning electron microscope (SEM, S-4300SE, Hitachi Co., Ltd., Tokyo, Japan) to identify a target coarse grain for the fabrication of the sample B micro-cantilever.

### 2.2. Methods

**Figure 1:**
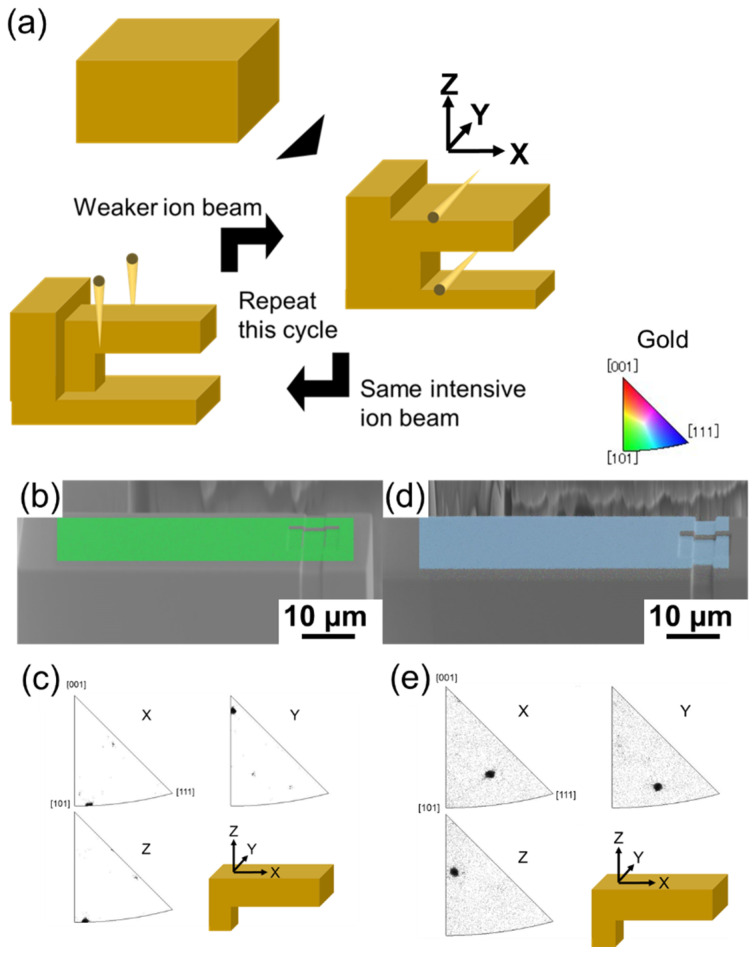
(**a**) FIB fabrication steps of the micro-cantilever, (**b**) EBSD map along the x-axis, (**c**) inverse pole figure of A-TW10, (**d**) EBSD map along the x-axis, and (**e**) inverse pole figure of B-TW10.

The micro-cantilevers were fabricated using a focused ion beam (FIB, FB2100, Hitachi, Tokyo, Japan) as shown in Figure 1a. To minimize the tapering effect [31], the ion beam was irradiated from the side of the micro-cantilever at a tilt angle of ±1.5°. Micro-cantilevers prepared from the Sanwa Kinzoku gold, with the loading direction parallel to the [1-10] orientation, the neutral plane parallel to the [110] orientation, an orientation error of less than 7° as determined from the EBSD results shown in Figure 1b,c, are referred to as sample A. In this study, 9 sample A micro-cantilevers were prepared, all with a length of 50 µm and variations in thicknesses and widths ranging from 5 to 15 µm. Additionally, another micro-cantilever, sample B, with its loading direction aligned parallel to the [0.37 −0.92 0.05] orientation and the neutral plane parallel to the [0.54 0.28 0.78] orientation as illustrated in Figure 1d,e, was prepared from the Nilaco gold. More detailed specifications of the 10 micro-cantilevers are provided in Table 1, with the sample names determined by the orientation, thickness, and width. The base cantilever was denoted as TW10, with a thickness and width of approximately 10 µm.

The bending test was performed using a machine specially designed for micro-samples developed in our group [32]. The test machine had a load resolution of 10 μN, and the displacement resolution was 5 nm. The strain rate was fixed at 0.125%/sec. Before and after the bending test, the samples were subjected to SEM and EBSD analysis. The moment arm, thickness, width, and length of the micro-cantilever for calculations of the stress and strain were measured from SEM observations.

## 3. Results and Discussion

**Figure 2:**
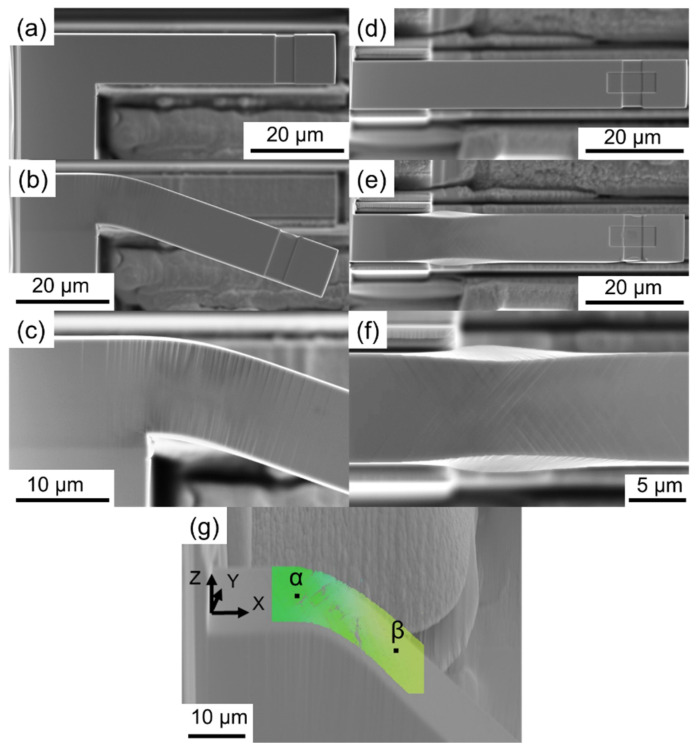
Side views of A-TW10: (**a**) as-fabricated, (**b**) deformed, and (**c**) support. Top views of A-TW10: (**d**) as-fabricated, (**e**) deformed, and (**f**) support. (**g**) EBSD mapping of the deformed A-TW10.

Figure 2 shows SEM images of the side and top views of the A-TW10 micro-cantilever before and after the bending test. It is evident from the observed slip lines in the side view that stress and deformations were concentrated at the support of the micro-cantilever. An enlarged side view image near the support is shown in Figure 2c. The top views depicted in Figure 2d–f illustrate necking and barreling deformations occurring at the support after the bending test. A closer look at the top view (Figure 2f) reveals necking deformation and slip lines on the top surface, while the bottom part exhibits barreling deformation. Similar deformation behaviors were observed among all nine sample A micro-cantilevers, with each showing local deformation at the support.

The development of slip lines in accordance with Schmid’s law is expected to occur on the active slip plane. Table 2 presents the Schmid factors calculated with [110] as the load direction, indicating that the active slip planes are (111) and (11-1). The angle between these planes is approximately 70°, which closely matches the angle between the observed slip lines in Figure 2f. This confirms that the slip lines followed the active slip plane as per Schmid’s law. Additionally, the EBSD result of A-T12.5W10 after the bending test, shown in Figure 2g, indicates deformation with crystal rotation. The difference in the angle between α and β was estimated to be 16°.

**Figure 3:**
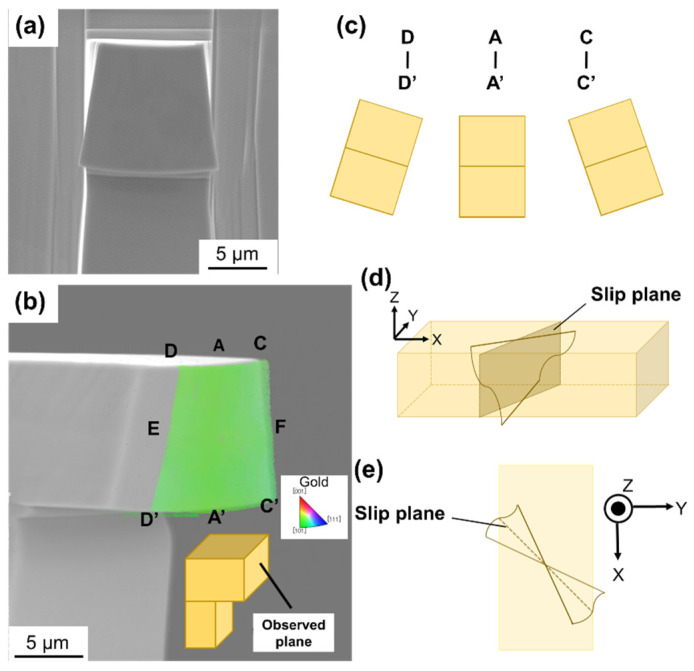
(**a**) SEM image and (**b**) EDSD analysis of the cross-section of A-TW10. (**c**) Illustration of necking and barreling deformations. (**d**) Side view and (**e**) top view illustrations of the rotation of the slip plane.

The cross-section of the deformed A-TW10 near the support, where the deformation was concentrated, was prepared as depicted in Figure 3a. The EBSD result of the cross-section is shown in Figure 3b. The misorientation angle along the width direction, from point E to point F, was approximately 12° to 15°, while the misorientation angle along the thickness direction, such as from point A to point A’, was relatively small and did not exhibit characteristic angle changes. This indicates that necking and barreling occurred due to crystal rotation, as illustrated in Figure 3c. 

In general, during deformation, the slip plane rotates in a direction parallel to the loading direction in tensile tests, and in a direction perpendicular to the loading direction in compression tests [33]. In bending tests, where both tensile and compression stresses exist, the slip plane rotates parallel to the loading direction around the top surface, while simultaneously rotating perpendicular to the loading direction around the bottom surface. These rotations on the top and bottom surfaces are depicted in Figure 3d. In this study, the active slip planes (111) and (11-1) are symmetrical to the X axis, resulting in necking and barreling occurring without distortion, as shown in Figure 3e.

**Figure 4:**
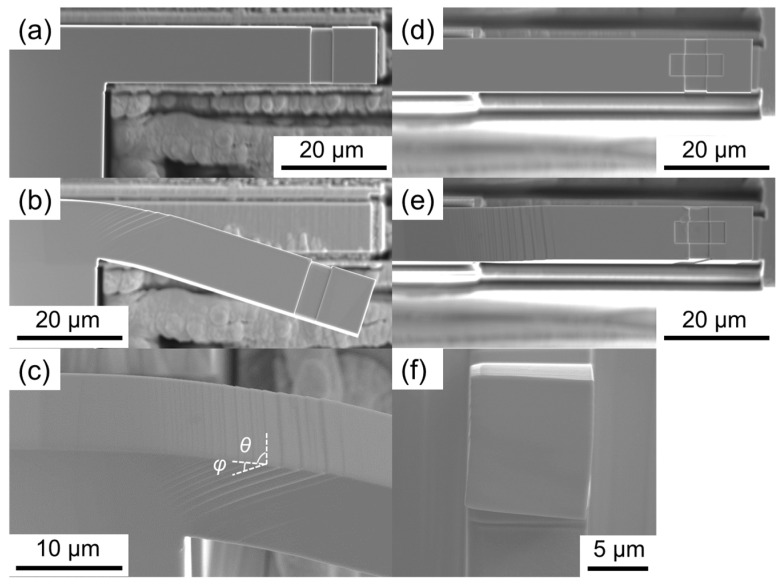
Side views of B-TW10: (**a**) as-fabricated, (**b**) deformed, and (**c**) near the support. Top views of B-TW10: (**d**) as-fabricated and (**e**) deformed. (**f**) Cross-section near the support of B-TW10.

Figure 4 shows SEM images of the B-TW10 before and after the bending test. The images reveal that deformations are concentrated near the micro-cantilever support, with noticeable slip lines observed, as shown in the enlarged view in Figure 4e. The angles of θ and φ in Figure 4e were measured as 86° and 36°, respectively. Slip trace analysis confirmed that the activated slip system follows Schmid’s law, with the corresponding Schmid factors presented in Table 3. Unlike the cantilever in the nine sample A micro-cantilevers, the B-TW10 did not exhibit necking and barreling deformation behaviors, as revealed in Figure 4f. This suggests that in single-slip deformation, the force for a slip plane to rotate is not as strong due to only a few slip planes being activated; thus, crystal rotation did not occur in the B-TW10. These results indicate that the deformation behavior in the bending test of a micro-cantilever is influenced by the crystal orientation.

The yield stresses were determined from the engineering stress–engineering strain curve obtained from the bending test and using the 0.2% strain threshold method [29,34]. The results are summarized in Table 1. It was observed that the yield stress of the sample B micro-cantilever (B-TW10) was significantly lower at 77 MPa compared to the range of 175~237 MPa for all nine sample A micro-cantilevers. This difference is likely attributed to the different number of activated slip planes, with sample A exhibiting multiple slip planes and a higher density of geometrically necessary dislocations (GNDs) compared to sample B. Also, sample B was fabricated from a heat-treated specimen. The internal stress and dislocation density in sample B could be lower than those in sample A, and these factors could contribute to the reduced yield stress.

**Figure 5:**
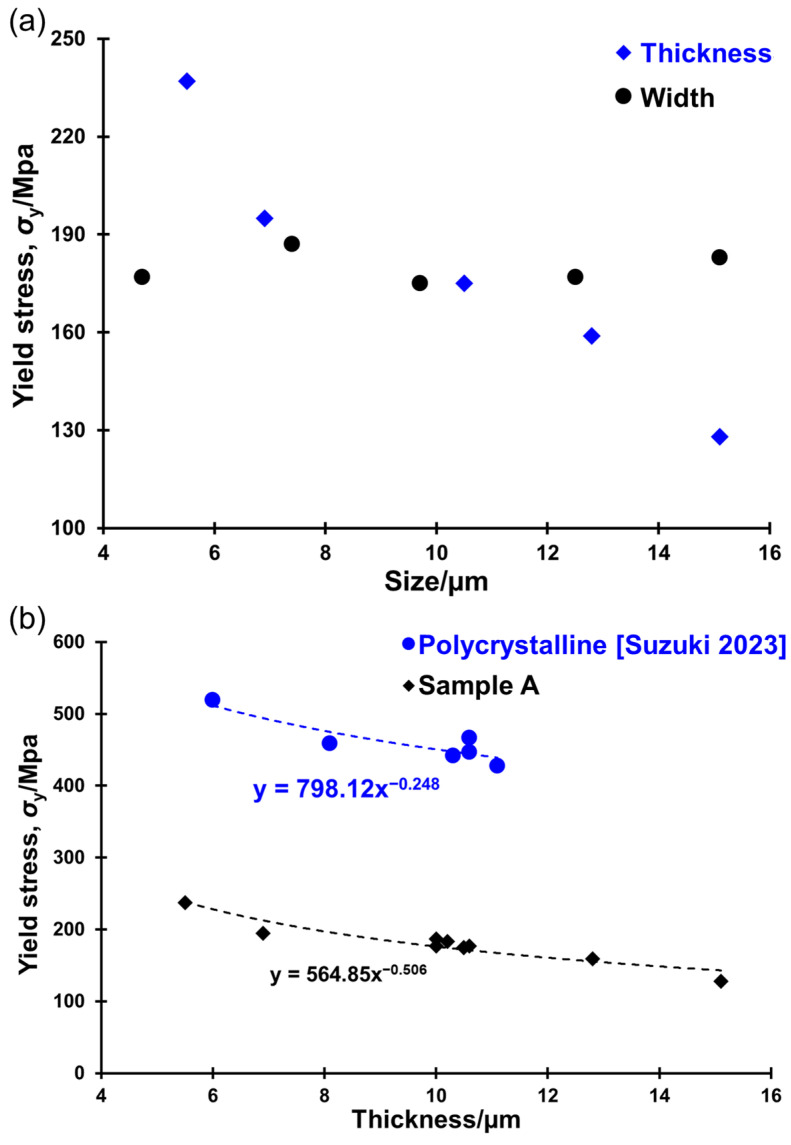
Plots of (**a**) yield stress versus width or thickness for sample A micro-cantilevers, and (**b**) yield stress versus thickness for polycrystal [29] and sample A micro-cantilevers.

As shown in Figure 5a, the yield stress was found to increase as the thickness decreased, while it did not show significant change with the reduction in width. This indicates that the sample size effect was only noticeable when altering the thickness, not the width. These results validate that the bending mechanical properties of micro-cantilevers are influenced by sample geometry, referred to as the sample geometry effect.

Comparing the sample geometry effect observed in polycrystal gold micro-cantilevers from a previous study [29] with the results from this study, a power law fit of the yield stress versus thickness is shown in Figure 5b. The power exponent was −0.506 for sample A in this study and −0.248 for polycrystals. This analysis demonstrates that single-crystal gold exhibits a stronger sample geometry effect than polycrystal gold. This higher dependency in single-crystal metals is consistent with the sample size effect reported by Greer et al. [18].

There are various theories that explain the effect of sample size under different conditions. In the bending of micro-cantilevers, factors such as strain gradient, dislocation interaction, pile up of dislocations on the neutral plane, and the reduction in dislocation sources have been discussed in previous studies [35,36]. According to this model, dislocations would be emitted from activated sources around the top/bottom surface, where shear stress is strong, and pile up on the neutral plane, where shear stress is weak. With fewer slip planes available to accommodate the geometrically necessary dislocations caused by the strain gradient due to the reduction in dislocation sources with smaller sample sizes, the mechanical strength increases.

In addition, the dislocation starvation model [17] becomes relevant under conditions without a strain gradient. This model suggests that when the sample size is small enough, it becomes easier for mobile dislocations to escape to the free surface rather than being pinned by other dislocations. Consequently, plasticity is accommodated by the nucleation of new dislocations, leading to an increase in mechanical strength under dislocation starvation. However, the current study indicates that the yield stress does not show a dependency on the width of the micro-cantilever. This suggests that the dislocation starvation model may not be the dominant factor in this case.

Instead, if the pile up of dislocations on the neutral plane and the reduction in dislocation sources are dominant, the yield stress is influenced by the distance between the top/bottom surface and the neutral plane of the micro-cantilever. This is because dislocations pile up quickly on the neutral plane with fewer slip planes when that distance decreases. Furthermore, this distance would depend on the thickness of a micro-cantilever, rather than the width. Therefore, it can be concluded that the pile up of dislocations on the neutral plane and the reduction in dislocation sources are important mechanisms contributing to the sample size effect in this case, which explains why the sample geometry effect occurs.

## 4. Conclusions

Gold materials used as miniaturized components in electronic devices, such as micro-components in MEMS devices, may have different geometries and crystalline characteristics. This study aims to investigate the interrelationship between the sample size effect, sample geometry effect, and crystalline orientation. In conclusion, the impact of sample geometry on bending behavior was investigated through bending tests on micro-cantilevers of varying widths and thicknesses. These micro-cantilevers, made from single-crystal pure gold using FIB fabrication, exhibited necking deformation on the top surface and barreling deformation on the bottom surface when loaded parallel to the [1-10] orientation with the neutral plane parallel to the [110] orientation. These simultaneous necking and barreling behaviors were not observed in a micro-cantilever with a single active slip plane. Furthermore, the yield stresses of the micro-cantilevers increased as the thickness decreased, while changes in width had minimal impact on the yield stress. These findings confirm that the sample size effect is influenced by sample geometry in bending, attributed to dislocation pile up on the neutral plane and the reduction in dislocation sources due to sample size reduction. This sample geometry effect has significant implications for the design of micro-components in MEMS devices.

## Figures and Tables

**Table 1:** Information about the micro-cantilevers.

Sample	Thickness (μm)	Width (μm)	Yield Stress (MPa)
A-T5W10	5.5	9.8	237
A-T7.5W10	6.9	9.7	195
A-TW10	10.5	9.7	175
A-T12.5W10	12.8	9.8	159
A-T15W10	15.1	9.3	128
A-T10W5	10.0	4.7	177
A-T10W7.5	10.0	7.4	187
A-T10W12.5	10.6	12.5	177
A-T10W15	10.2	15.1	183
B-TW10	10.5	10.1	77

**Table 2:** Schmid’s factor with [110] as the loading axis.

Slip System	Schmid’s Factor (Absolute Value)
(111)/[$1\bar{1}0$]	0
(111)/[$10\bar{1}$]	0.408
(111)/[$01\bar{1}$]	0.408
($11\bar{1}$)/[$1\bar{1}0$]	0
($11\bar{1}$)/[101]	0.408
($11\bar{1}$)/[011]	0.408
($1\bar{1}1$)/[110]	0
($1\bar{1}1$)/[$10\bar{1}$]	0
($1\bar{1}1$)/[011]	0
($\bar{1}11$)/[110]	0
($\bar{1}11$)/[101]	0
($\bar{1}11$)/[$01\bar{1}$]	0

**Table 3:** Schmid’s factor with [0.54 0.28 0.78] as the loading axis.

Slip System	Schmid’s Factor (Absolute Value)
(111)/[$1\bar{1}0$]	0.17
(111)/[$10\bar{1}$]	0.16
(111)/[$01\bar{1}$]	0.33
($11\bar{1}$)/[$1\bar{1}0$]	0.004
($11\bar{1}$)/[101]	0.02
($11\bar{1}$)/[011]	0.016
($1\bar{1}1$)/[110]	0.35
($1\bar{1}1$)/[$10\bar{1}$]	0.10
($1\bar{1}1$)/[011]	0.46
($\bar{1}11$)/[110]	0.17
($\bar{1}11$)/[101]	0.28
($\bar{1}11$)/[$01\bar{1}$]	0.10

## Data Availability

The data that support the findings of this study are available from the corresponding author upon reasonable request.
